# Retention in Care Trajectories of HIV-Positive Individuals Participating in a Universal Test-and-Treat Program in Rural South Africa (ANRS 12249 TasP Trial)

**DOI:** 10.1097/QAI.0000000000001938

**Published:** 2018-12-18

**Authors:** Andréa Gosset, Camelia Protopopescu, Joseph Larmarange, Joanna Orne-Gliemann, Nuala McGrath, Deenan Pillay, François Dabis, Collins Iwuji, Sylvie Boyer

**Affiliations:** a INSERM, IRD, SESSTIM, Sciences Economiques & Sociales de la Santé, Traitement de l'Information Médicale, Aix Marseille University, Marseille, France;; b ORS PACA, Observatoire régional de la santé Provence-Alpes-Côte d'Azur, Marseille, France;; c Africa Health Research Institute, KwaZulu-Natal, South Africa;; d Ceped, Institut de Recherche pour le Développement, Université Paris Descartes, Inserm, Paris, France;; e Inserm, UMR 1219, Bordeaux Population Health Research Center, University Bordeaux, Bordeaux, France;; f Inserm, UMR 1219, ISPED, Bordeaux Population Health Research Center, Bordeaux, France;; g Faculty of Medicine and Faculty of Human, Social and Mathematical Sciences, University of Southampton, United Kingdom;; h Research Department of Infection and Population Health, University College London, London, United Kingdom;; i Division of Infection and Immunity, University College London, London, United Kingdom; and; j Department of Global Health & Infection, Brighton and Sussex Medical School, Brighton, United Kingdom.

**Keywords:** universal test and treat, HIV, South Africa, retention in care, trajectories

## Abstract

**Objective:**

To study retention in care (RIC) trajectories and associated factors in patients eligible for antiretroviral therapy (ART) in a universal test-and-treat setting (TasP trial, South Africa, 2012–2016).

**Design:**

A cluster-randomized trial whereby individuals identified HIV positive after home-based testing were invited to initiate ART immediately (intervention) or following national guidelines (control).

**Methods:**

Exiting care was defined as ≥3 months late for a clinic appointment, transferring elsewhere, or death. Group-based trajectory modeling was performed to estimate RIC trajectories over 18 months and associated factors in 777 ART-eligible patients.

**Results:**

Four RIC trajectory groups were identified: (1) group 1 “remained” in care (reference, n = 554, 71.3%), (2) group 2 exited care then “returned” after [median (interquartile range)] 4 (3–9) months (n = 40, 5.2%), (3) group 3 “exited care rapidly” [after 4 (4–6) months, n = 98, 12.6%], and (4) group 4 “exited care later” [after 11 (9–13) months, n = 85, 10.9%]. Group 2 patients were less likely to have initiated ART within 1 month and more likely to be male, young (<29 years), without a regular partner, and to have a CD4 count >350 cells/mm^3^. Group 3 patients were more likely to be women without social support, newly diagnosed, young, and less likely to have initiated ART within 1 month. Group 4 patients were more likely to be newly diagnosed and aged 39 years or younger.

**Conclusions:**

High CD4 counts at care initiation were not associated with a higher risk of exiting care. Prompt ART initiation and special support for young and newly diagnosed patients with HIV are needed to maximize RIC.

## Abstract 2

Supplemental Digital Content is Available in the Text.

## INTRODUCTION

South Africa has the highest number of people living with HIV (PLWHIV) in the world, estimated at 7 million in 2015.^1^ Forty-nine percent receive antiretroviral therapy (ART), making it the largest treatment program worldwide.^2^ Despite a reduction in HIV-related morbidity and mortality and a consequent increase in life expectancy,^3^ HIV incidence remains unacceptably high.^4^

In 2016, South Africa adopted the WHO's recommendation to implement a universal test-and-treat (UTT) strategy for HIV.^4^ The success of this strategy depends on sustained retention in care (RIC).^5,6^ Modeling estimated that to achieve an HIV incidence rate below 0.1% per year by 2050, rates of ART coverage and RIC need to reach 95%.^5^

A meta-analysis in 2015 estimated that RIC among adults who initiated ART in South Africa was 77% at 12 months and 75% at 24 months.^7^ In 2017, the South African government set the objective of reaching a retention rate of 90% at 12 months after ART initiation among PLWHIV by 2018/2019, increasing to 95% by 2021/2022.^4^

To achieve this ambitious target, a greater understanding of the barriers to RIC in UTT settings, where PLWHIV start treatment early, is needed. To date, the literature in low- and middle-income countries has mainly focused on non-RIC among pre-ART patients^8–10^ or patients who start ART with low CD4 counts (≤350 cells/mm^3^) and/or at the AIDS stage.^11–13^ Evidence suggests a lower RIC rate among pre-ART patients with high CD4 counts,^9,10,14^ but it is still unknown whether high CD4 counts (>350 cells/mm^3^) at ART initiation will improve or deteriorate RIC. In the only study conducted to date in a UTT setting—the SEARCH trial in Uganda and Kenya—the authors found high RIC among patients with high CD4 counts (350–500 cells/mm^3^ and >500 cells/mm^3^).^15^ However, concerns remain that patients with high CD4 counts may be more reluctant to engage in treatment.^16^ Moreover, one limitation of previous RIC studies is the assumption that patients follow a single-care trajectory, whereas, in reality, patients can cycle in and out of care, and so multiple trajectories are possible.^17,18^

In this study, we aimed to study RIC trajectories and associated factors in ART-eligible patients enrolled in the UTT TASP trial ANRS 12249 implemented in rural South Africa.

## METHODS

### Study Setting and Design

ANRS 12249 TasP (Treatment as Prevention) trial was a cluster-randomized trial conducted between 2012 and 2016 in the Hlabisa subdistrict, KwaZulu-Natal, in South Africa. The area is mainly rural with scattered homesteads. It is also among the most exposed to HIV in the country^19^ with an estimated 30% HIV prevalence in adults (15–49 years).^20^ The main objective of the trial was to investigate whether HIV testing of all adult populations followed by immediate ART initiation for all those testing positive (irrespective of immunological status or clinical stage) would reduce HIV incidence in this area.

The trial protocol is described elsewhere.^21,22^ Briefly, it was implemented in 22 (11 intervention and 11 control) geographic clusters, each with an average population of 1000 residents 16 years or older. In all clusters, home-based counseling and HIV testing (HBHT) were offered every 6 months to all eligible household members, that is, residents 16 years or older. Individuals testing HIV positive were then referred to their cluster trial clinic. These clinics, which were set up specifically for the trial, were located <5 km from their homes. The clinics in the intervention clusters immediately offered ART to all PLWHIV, regardless of CD4 count or clinical stage. Instead, PLWHIV in the control clinics initiated ART according to the eligibility criteria defined by national guidelines: CD4 count ≤350 cells/mm^3^, WHO stage 3/4, and/or pregnancy.^23^ In January 2015, these criteria were extended to include CD4 count ≤500 cells/mm^3^, hepatitis B positivity, and HIV-negative partners in serodiscordant relationships.^24^ In all the trial clinics, patients who initiated ART had monthly clinical follow-up visits, whereas pre-ART patients had a quarterly clinical follow-up. All patients, whether pre-ART or ART-treated, who were more than 3 months late for an appointment in their clinic, were contacted by phone or during home-based visits. HIV care was also available in government clinics located in the trial area, which also provided care to non-HIV patients.^25^ On request, participants could transfer out from trial care to one of these clinics, in or outside the trial area.

The Biomedical Research Ethics Committee (BREC) of KwaZulu-Natal University (BFC 104/11) and the South African Medicines Control Council approved the trial. All participants provided written informed consent.

### Outcome

The study outcome was a time-varying binary variable “retention in trial care” (RIC) status, describing whether a patient remained or not in trial care during the 18-month study period. A patient was considered to have exited trial care if she/he was >3 months late for his/her last appointment at the clinic, if she/he transferred out, or if she/he died. RIC status in the trial clinics was assessed for each patient every month from 4 to 18 months after his/her baseline visit (RIC status was therefore not defined during the first 4 months of follow-up). A patient lost to follow-up (LTFU) at a given month could reenter trial care if she/he revisited a trial clinic later.

### Study Population and Study Period

The study population included HIV-positive individuals eligible to initiate ART (as per the trial protocol) at their first visit in one of the trial's clinics (baseline visit), who had their baseline visit ≥18 months before the end of the trial (June 30, 2016), and who did not die in the first 4 months of follow-up. The study period covered from 4 to 18 months after the baseline visit of each patient.

### Covariates

Information on covariates used in the analysis was obtained from (1) face-to-face questionnaires administered during home-based visits and at baseline visit in clinics, and (2) clinical report forms completed by caregivers at baseline and during follow-up.

Covariate information collected during home-based visits included gender, age, education, having children, occupation, household wealth, and geographical accessibility to the trial's clinics. Covariates collected at the baseline clinic visit included CD4 count, having a regular partner, social support, psychological distress (Patient Health Questionnaire-4 scale^26^), time between referral and baseline visit, and being newly diagnosed at referral (ie, reporting—during HBHT—no previous HIV-positive diagnosis and not being registered as a patient with HIV in local government clinics). We also distinguished patients who initiated ART within 1 month after baseline from those who did not. Finally, we classified the 22 clusters into a binary variable: (1) clusters with low number of patients (13–155) followed in the trial's clinics (HIV prevalence in those clusters was 17.5%–35.4%) and (2) clusters with high number of patients (212–422) followed in the trial's clinics (HIV prevalence: 32.3%–39.4%).

### Statistical Analysis

Group-based trajectory modeling (GBTM) was performed to estimate RIC trajectories during the study period using the outcome variable “retention in trial care.” GBTM is a semiparametric mixture modeling procedure for longitudinal data,^27^ which identifies trajectory groups over time. It classifies individuals into groups with similar evolution for the outcome variable and identifies factors associated with these groups.

The optimal number of trajectory groups was evaluated using the Bayesian Information Criterion, by selecting the number of groups that best represented the heterogeneity between the trajectories.

The probabilities of group membership were estimated using a multinomial logistic model. Patients were assigned to the group for which they had the highest estimated probability of membership. Each identified group had a specific trajectory that illustrated the probabilities of having exited care at a given month from 4 to 18 months after baseline. We assumed that the probability of exiting care followed a binary logit distribution.

Factors associated with trajectory group membership were tested for in the analysis as fixed covariates measured at the baseline visit and at 1 month after baseline for the ART initiation covariate. The model parameters were estimated by the maximum likelihood method.

Covariates were considered eligible for the GBTM multivariable model if their association with group membership indicated a *P* value <0.20 in GBTM univariable analyses. A forward stepwise procedure was used to select the covariates in the final multivariable model with a *P* value ≤0.05.

All analyses were performed using Stata/SE 12.1 for Windows software.^28^

### Sensitivity Analysis

Sensitivity analysis was conducted to assess the robustness of the results when considering the following: (1) a longer follow-up period (ie, from 4 to 24 months after baseline) and (2) alternative hypotheses for transfers-out. Specifically, we considered transfers-out as missing data from the time the patients transferred out (accordingly, exiting trial care only included deaths and LTFU). Second, we assessed an optimistic but realistic scenario where transfers-out were considered to be “retained in care.”

## RESULTS

### Cohort Profile

**FIGURE 1.**
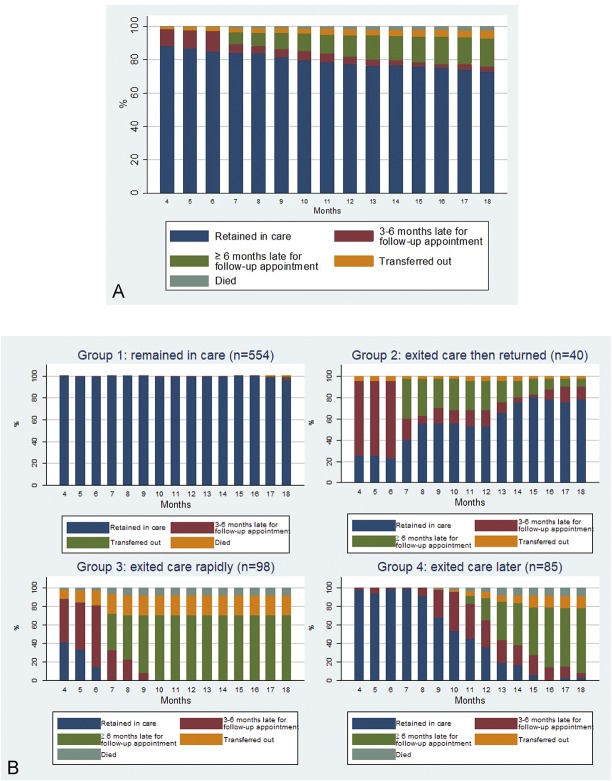
RIC status of ART-eligible patients at the first clinic visit from 4 to 18 months of clinical follow-up, overall (A) and according to trajectory groups (B) (ANRS 12249 TasP trial, n = 777).

Of the 7647 PLWHIV who were referred to the trial clinics over the trial period, 3019 (39.5%) actually visited a trial clinic at least once. Among these, 1412 (46.8%) were already on ART at the baseline visit, 428 (14.2%) were not eligible for ART, and 16 (0.5%) had missing data for either ART status or CD4 cell count. Of the remaining 1163 (38.5%) individuals—all eligible to initiate ART at baseline—we retained those who had their first visit ≥18 months before the end of the trial (788 patients), and excluded those who died during the first 4 months of follow-up (10 patients) because retention was not defined during this period, as well as one patient whose recorded date of death was inconsistent. Our study population therefore comprised 777 ART-eligible patients (see Figure 1, Supplemental Digital Content, http://links.lww.com/QAI/B264).

Approximately two-thirds (70.7%) of our study population were women (Table 1). The median age [interquartile range (IQR)] at baseline was 35 (27.5–46.6) years, and 76.2% had a regular partner. Most patients (88.5%) were already diagnosed HIV positive at referral. Two-thirds (66.3%) entered HIV care at one of the trial's clinics within 1 month after referral, and 40% resided <1 km from their clinic. Over a quarter (26.3%) of patients had a CD4 count >500 cells/mm^3^ at baseline, and 54% initiated ART within 1 month.

**TABLE 1.**
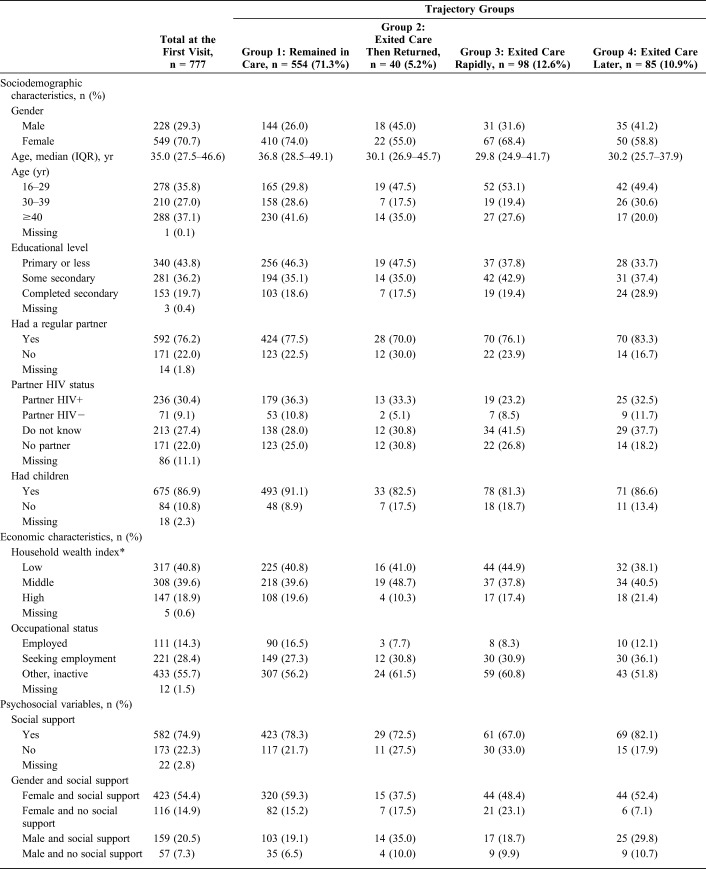

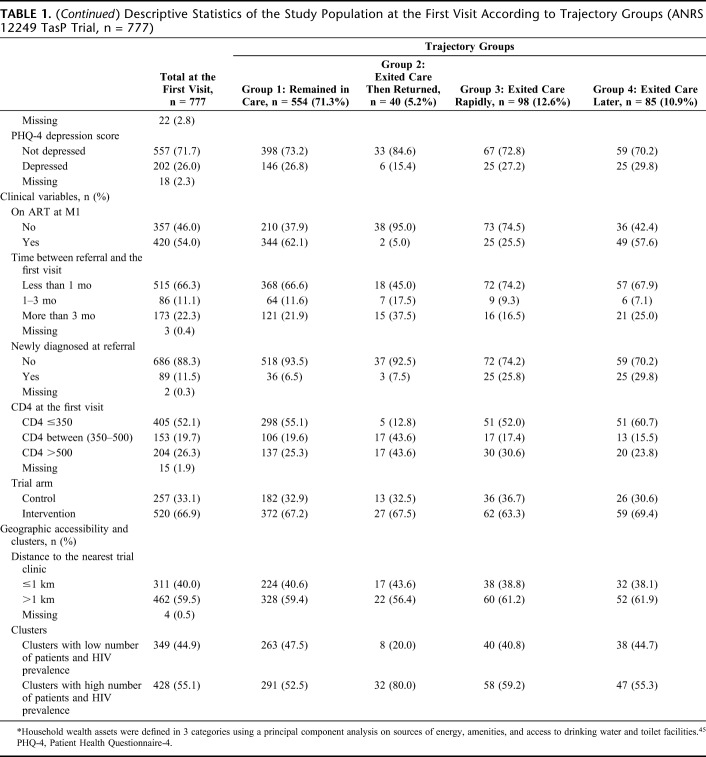
Descriptive Statistics of the Study Population at the First Visit According to Trajectory Groups (ANRS 12249 TasP Trial, n = 777)

### RIC and Retention Trajectories

The overall RIC rate was 77.5% at 12 months (M12) and 72.8% at M18 (Fig. 1A). Among patients exiting trial care, LTFU was the main cause of attrition (76.6% and 73.4% at M12 and M18, respectively), whereas death accounted for 6.9% and 8.1%, respectively, and transfers-out for 16.6% and 18.5%. The median (IQR) follow-up duration before exiting care for the first time was 7 (4–11) months.

RIC rates at M18 were similar in both arms (70.8%—control versus 73.8%—intervention, *P* = 0.37), and between the 3 different CD4 count categories (71.9%, 77.8%, and 69.6% for CD4 counts ≤350 cells/mm^3^, 350–500 cells/mm^3^, and >500 cells/mm^3^, respectively; *P* = 0.22). In addition, focusing only on the 704 (90.6%) patients who initiated ART over the study period, the RIC rate at M18 reached 80.0% (79.4%—control versus 80.3%—intervention, *P* = 0.79).

**FIGURE 2.**
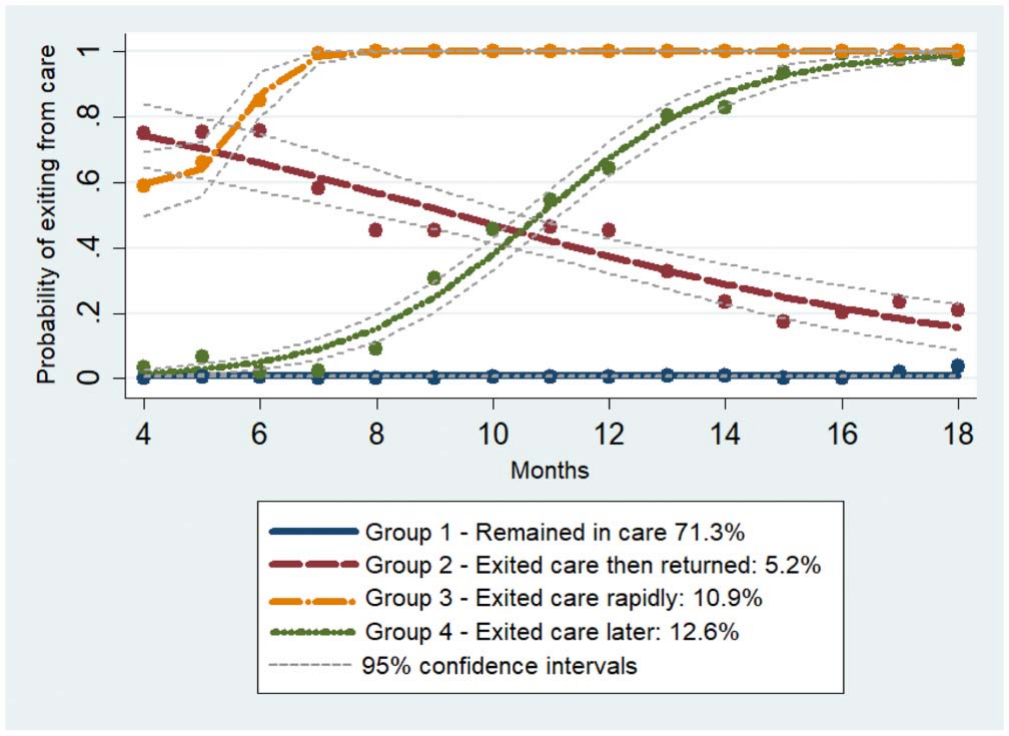
Care trajectories in trial clinics over 18 months of clinical follow-up among patients eligible for ART initiation at the first visit (ANRS 12249 TasP trial, n = 777).

Four different trajectories were identified (Fig. 2). Group 1 patients (71.3% of the study population) “remained in care” throughout the study period. At M18, less than 1% of them had died or transferred out (Fig. 1B). Group 2 patients (5.2%) exited care and then returned later, after a median time (IQR) of 4 (3–9) months (hereafter the “returned” group). At M18, no deaths had occurred in this group, and only one patient (2.5%) had transferred out. Group 3 patients (12.6%) “rapidly exited” care after a median time (IQR) of 4 (4–6) months of follow-up. In this group, all patients had exited trial care at M18 (8.2% had died and 21.4% had transferred out). Finally, group 4 patients (10.9%) “exited care later” after a median time (IQR) of 11 (9–13) months of follow-up. At M18, 9.4% of them had died, whereas 12.9% had transferred out.

### ART Initiation by the Trajectory Group

Although all study patients were ART eligible at baseline, overall 90.6% initiated ART during the study period. Furthermore, ART initiation differed widely across the 4 trajectory groups (Table 2). In groups 1 and 4, a large majority of patients initiated ART during the study period (99.6% and 87.1%, respectively), mainly during the first month after baseline. In group 2, a large majority (85.0%) also initiated ART during the study period but after a longer delay [median (IQR) time after baseline: 343 (208–449) days]. Conversely, in group 3, only 44.9% initiated ART during the study period but within a short delay after baseline [median (IQR) time: 27.5 (15.5–49.5) days].

**TABLE 2.**
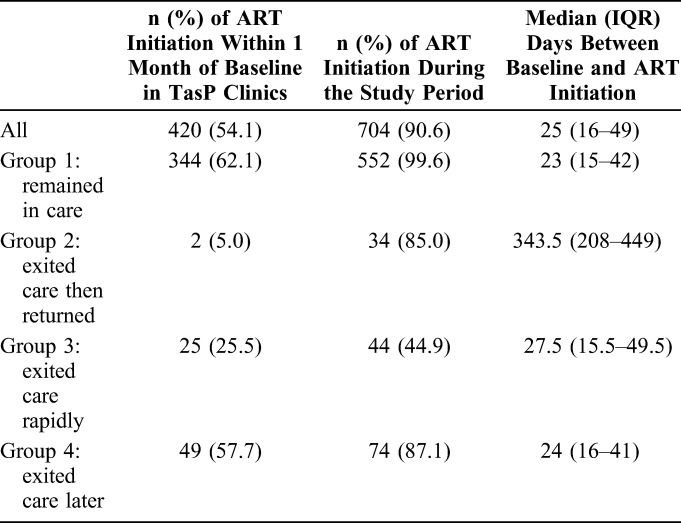
Patients Who Initiated ART Among the Study Population According to Trajectory Groups (ANRS 12249 TasP Trial, n = 777)

### Factors Associated With Trajectory Groups

Table 3 presents the results of the univariable and multivariable analyses.

**TABLE 3.**
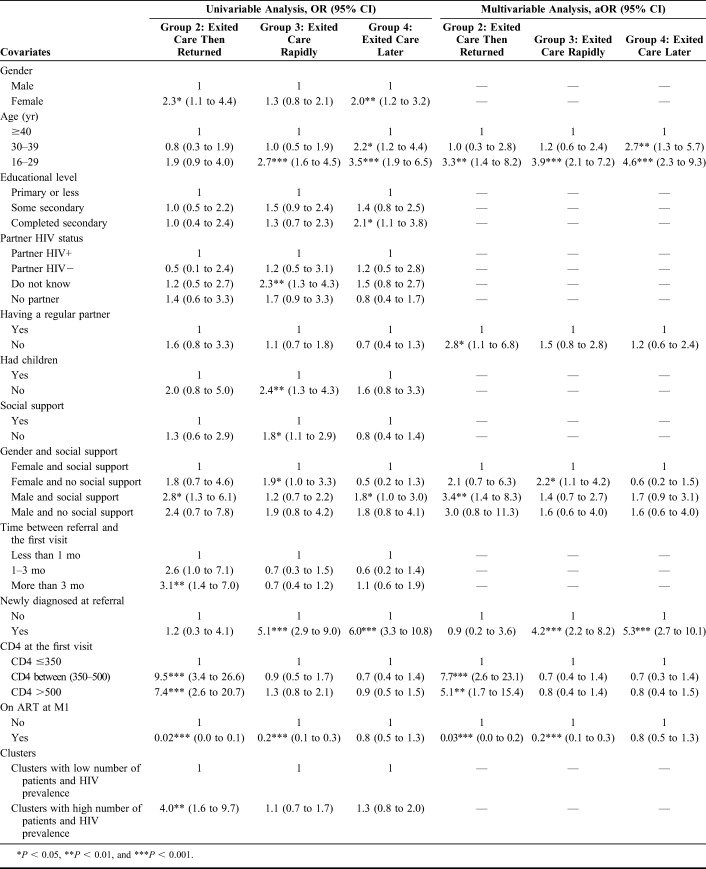
Factors Associated With Trajectory Groups (Reference = Group 1: Remained in Care), Univariable and Multivariable Analyses (ANRS 12249 TasP Trial)

In the multivariable model, the patients of group 2 compared with those in group 1 (reference group) were more likely to be young {adjusted odds ratio (aOR) [95% confidence interval (CI)] = 3.3 (1.4 to 8.2) for 16–29 years old versus ≥40 years old}, without regular partner [2.8 (1.1–6.8)], men receiving social support [3.4 (1.4–8.3) versus women receiving social support], and to have high CD4 counts [7.7 (2.6–23.1) and 5.1 (1.7–15.4) for CD4 counts between 350 and 500 cells/mm^3^ and >500 cells/mm^3^, respectively, versus CD4 counts ≤350 cells/mm^3^].

The patients in group 3, compared with those in group 1, were significantly younger [3.9 (2.1–7.2) for patients aged 16–29 years old versus ≥40 years old], were more likely to be women without social support [2.2 (1.1–4.2) versus women with social support], and newly diagnosed [4.2 (2.2–8.2)].

By contrast, the patients in group 2 and those in group 3, compared with those in group 1, were less likely to have initiated ART within 1 month after baseline [0.03 (0.0–0.2) and 0.2 (0.1–0.3), respectively].

Finally, the patients in group 4, compared with those in group 1, were more likely to be young [4.6 (2.3–9.3) for 16–29 years old and 2.7 (1.3–5.7) for 30–39 years old versus ≥40 years old] and newly diagnosed [5.3 (2.7–10.1)].

### Sensitivity Analyses

When estimating the trajectory groups over a 24-month period (n = 536), the retention rate decreased to 69.2% at M24 and was similar in both arms (63.9%—control versus 71.4%—intervention, *P* = 0.09), and between the 3 CD4 count categories at baseline (69.8%, 74.7%, and 65.6% for CD4 counts ≤350 cells/mm^3^, between 350 and 500 cells/mm^3^, and >500 cells/mm^3^, respectively; *P* = 0.311). A similar pattern including 4 trajectory groups was identified, but an additional group of patients (group 5) who exited care after a median (IQR) time of 17 (15–20) months emerged (see Figure 2, Supplemental Digital Content, http://links.lww.com/QAI/B264). Group 5 included 41 (7.6%) patients who were all in group 4 of the main analysis (over the 18-month period). The only factor associated with group 5 was being a woman without social support [aOR (95% CI) = 2.6 (1.1 to 6.3) versus a woman reporting social support], whereas associated factors for the 4 other groups were the same as those identified in the main analysis.

When considering transfers-out as missing data, the retention rate at M12 and M18, respectively, increased to 80.5% and 76.7%. We found the same associated factors for each group as in the main analysis, except for social support, which was no longer significant (see Table 1, Supplemental Digital Content, http://links.lww.com/QAI/B264). Similar results were found when considering transfers-out as “remaining in care”: the RIC rate at M12 and M18, respectively, increased to 81.2% and 77.9%, and the same associated factors were identified (see Table 2, Supplemental Digital Content, http://links.lww.com/QAI/B264).

## DISCUSSION

This study investigated RIC among HIV-positive patients in Kwazulu-Natal in South Africa, who were eligible for ART in a UTT setting where HIV prevalence ranged from 17% in very rural areas to 39% in communities close to the zone's national highway.^29^ Retention at 18 months was 72.8% overall and 80.6% if we only consider patients who initiated ART during the study period. Furthermore, using an original approach—GBTM—we identified care trajectories and their respective associated factors in this population, which is central for tailoring and prioritizing interventions. We showed that patterns of engagement with care are not uniform. Although three quarters of the study patients remained in care during the whole study period, 3 trajectories for exiting care emerged. Two corresponded to patients who left care and did not return during the study period (12.6% exited care after a very short follow-up duration, whereas 10.9% left after a longer duration). The third trajectory (5.2%) represented patients who exited care relatively rapidly but then returned. Our findings also suggest that initiating care in a UTT setting is not associated with lower retention, but that patients with high CD4 counts are more likely to exit care and then return. In addition, prompt ART initiation (within 1 month after the first visit in a trial clinic) was associated with a lower risk of exiting care rapidly and of exiting then returning. The main factors associated with care exit trajectories (either rapidly or later) included male gender, young age, and being newly diagnosed.

Retention rates found in our study are slightly higher than those estimated for the same period among patients initiating ART in South Africa's national ART program (80.6% versus 71%). Although relatively high, these retention rates are still well below 95%, the estimated rate needed to ensure the eradication of the HIV epidemic^5^ and the target set by the 2017–2022 South African National Strategic Plan.^4^ In addition, we found no significant difference in retention rates between the trial's arms or the CD4 count categories (≤350; 350–500; and >500 cells/mm^3^) at baseline. This was confirmed in multivariable analysis where patients with high CD4 counts (350–500 cells/mm^3^ and >500 cells/mm^3^) were not at higher risk of exiting care (either rapidly or later) than those with CD4 counts ≤350 cells/mm^3^. These findings suggest that initiating ART early in UTT settings is not associated with lower retention, probably because immediate ART initiation limits the duration of the pre-ART period, when the risk of exiting care is the highest.^30^ However, we showed that patients with high CD4 counts had a higher risk of exiting care and returning afterward. In addition, ART initiation within 1 month after the first visit to a trial clinic was significantly associated with a lower risk of exiting care rapidly (whether subsequently returning or not), suggesting that in a UTT setting, rapid ART initiation fosters retention. Interestingly, in the “returned” group, despite relatively high ART uptake over 18 months (85%), almost 95% of the patients had not initiated ART within 1 month, but did so within approximately 1 year. Delayed ART initiation in those with high CD4 count may be due to patients being hesitant to initiate ART rapidly,^31^ but also due to care providers prioritizing patients with lower CD4 counts in clinics with high patient loads.^32^

As found in other settings,^33,34^ retaining young patients in care is a challenge. Indeed, young age (<30) was a common risk factor for the 3 trajectories of care exit. It has been shown that this population had more competing life activities preventing them from attending clinical appointments on a regular basis.^17,31,35^ The trial setting was also characterized by a high migration level, which may have contributed to lower engagement in care by younger individuals who are more mobile.^36,37^

Furthermore, our findings highlight the importance of providing support to newly diagnosed HIV-positive individuals and of closely accompanying them on the HIV care continuum. Indeed, in the TasP trial, these people were less likely to be linked to care^38^ and had a higher risk of exiting care. This not only suggests that a long delay is required to first accept the disease, and to decide whether or not to attend a clinic, but also that newly diagnosed persons who attend a clinic may not be ready to engage steadfastly in care. Although such difficulties are not specific to the UTT strategy,^39,40^ they may be more frequent in this setting because this strategy does not rely on a voluntary testing initiative, and therefore, people may be less psychologically prepared to receive a positive diagnosis.

In this rural area of Kwazulu-Natal in South Africa, where HIV prevalence has reached extremely high levels, interventions are urgently needed to accelerate access to ART and to optimize RIC, with the goal of achieving viral suppression in PLWHIV and reducing new infection incidence in the community. Prompt and early ART initiation proposed in a UTT setting may be an effective means to reach this objective. In the TasP trial, most of PLWHIV who initiated ART within 1 month had only one visit in a trial clinic before ART initiation. However, a non-negligible proportion of our study population (7.2%) never returned after their first visit, and a significant proportion of those who exited care during the study period (30.6%) attended clinics only once. Considering the importance of the first visit for future retention, a great deal of attention should be paid to patients during this visit, to adequately prepare them for ART initiation. Special attention is needed for the youngest, those newly diagnosed, and those with high CD4 counts who may be more hesitant to engage steadfastly in care and may require additional visits before initiating ART. Home-based ART initiation is another potential intervention, which may encourage rapid ART initiation if patients are adequately prepared.^41^

Our study has limitations. First, we focused on RIC only in the trial's clinics because we lacked information about the retention status of patients who transferred out to public or private facilities. The latter were assumed to have exited care, which may have led to an underestimation of the retention rate. However, sensitivity analyses showed that our results are robust when considering alternative hypotheses for transfers-out. Second, although a tracking team contacted patients LTFU either by phone or during home-based visits, a certain number of silent transfers may have occurred, contributing to an underestimation of the retention rate. This limitation has often been mentioned in other studies.^42,43^ Third, although the TasP trial has been implemented at the population level with HIV status ascertained for 83% of adults living in the trial area,^29^ only 39.5% (3019/7647) of HIV-positive individuals referred for HIV care during HBHT actually attended a trial clinic. However, a significant proportion (42.7%) of the 7647 participants were already in the care of government clinics. Most of the latter (approximately 95%) were already ART-treated and thus not eligible for our study. In addition, according to a previous study on linkage to care in the trial, the majority (ie, approximately 72%) of HIV-positive individuals not in care at referral were not linked to care at 3 months (either in TasP or in government clinics), whereas those linked to care attended the trial's clinics and not the government clinics.^44^ This suggests that selection bias is possible but should be limited, as the large majority (ie, 86%) of our target population (HIV-positive individuals who initiated care, ie, who were not already being treated) were included in the trial's clinics.

Despite these limitations, this study brings great added value to current knowledge about RIC in the context of UTT strategies in sub-Saharan Africa. Our approach to analyzing RIC is innovative and promising, as it does not consider retention as a simple binary variable at a given point of time, rather a dynamic phenomenon where patients can cycle in and out of care, with multiple possible trajectories. It highlights the different trajectories of disengagement from care and suggests that initiating care in a UTT setting is not associated with lower retention.

Our findings may also inform policy makers' decision on the strategies to improve RIC, which is crucial for maximizing the impact of ART on the reduction of incidence. This includes ensuring prompt ART initiation, and targeting young, newly diagnosed patients and those with high CD4 counts, in particular during initial follow-up visits.

## Supplementary Material

SUPPLEMENTARY MATERIAL
