# Supplementary material for: Retention in Care Trajectories of HIV-Positive Individuals Participating in a Universal Test-and-Treat Program in Rural South Africa (ANRS 12249 TasP Trial)
Source: J Acquir Immune Defic Syndr. 2018 Dec 18;80(4):375–85. doi: 10.1097/QAI.0000000000001938 (PMC6410969; doi:10.1097/QAI.0000000000001938)
Supplement: SUPPLEMENTARY MATERIAL [file qai-80-375-s001.docx]

**Supplemental Digital Content**

**Supplemental Figure 1: Flow-chart of the study population (ANRS 12249 TasP trial)**

**
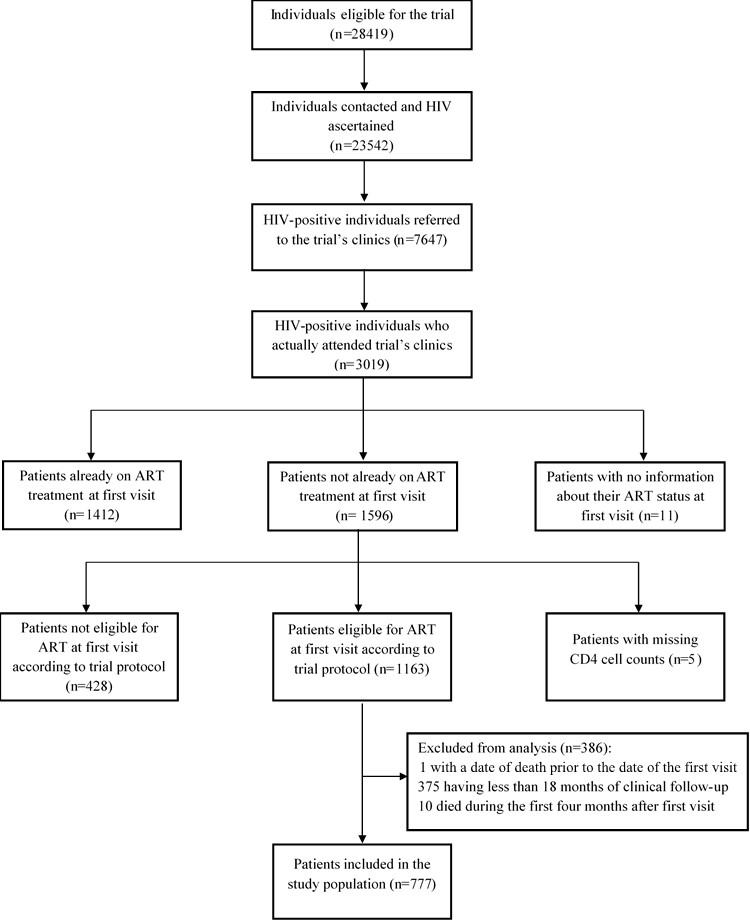
**

**Supplemental Figure 2: Care trajectories in trial clinics over 24 months of clinical follow-up among patients eligible for ART initiation at the first visit (ANRS 12249 TasP trial, n=536)**

**
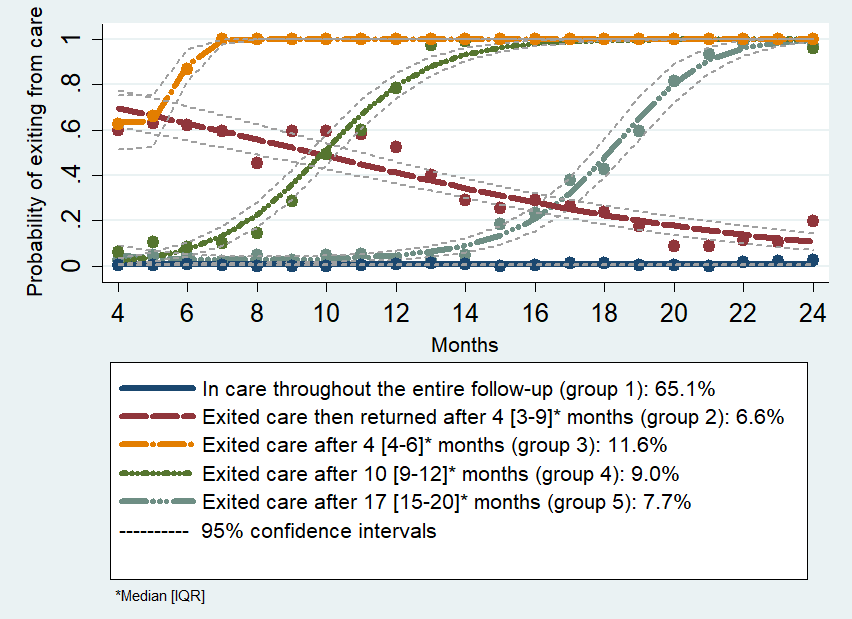
**

**Supplemental Table 1: Sensitivity analysis of factors associated with trajectory groups (reference= Group 1 “Remained in care”) when patients who transferred out were considered as missing, multivariable analysis (ANRS 12249 TasP trial, n=725)**

|  | **Groups** | | | |
| --- | --- | --- | --- | --- |
| **Covariates– aOR [95% CI]** | **Group 2: exited care then returned** | **Group 3: exited care rapidly** | **Group 4: exited care later** |  |
| *Gender & Social support* |  |  |  |  |
| Female & social support | 1 | 1 | 1 |  |
| Female & no social support | 1.5 [0.5,4.4] | 1.9 [0.8,4.1] | 0.6 [0.2,1.6] |  |
| Male & social support | 3.3^**^ [1.4,7.9] | 1.8 [0.9,3.7] | 1.8 [0.9,3.4] |  |
| Male & no social support | 2.3 [0.5,9.9] | 1.9 [0.7,5.0] | 1.9 [0.7,4.7] |  |
| *Age (years)* |  |  |  |  |
| ≥40 | 1 | 1 | 1 |  |
| 30-39 | 1.0 [0.4,3.0] | 1.0 [0.4,2.3] | 2.9^**^ [1.3,6.4] |  |
| 16-29 | 3.1^*^ [1.3,7.7] | 3.6^***^ [1.8,7.2] | 4.5^***^ [2.1,9.6] |  |
| *Having a regular partner* |  |  |  |  |
| Yes | 1 | 1 | 1 |  |
| No | 3.3^*^ [1.3,8.1] | 1.5 [0.7,3.0] | 1.1 [0.5,2.4] |  |
| *Newly diagnosed at referral* |  |  |  |  |
| No | 1 | 1 | 1 |  |
| Yes | 1.1 [0.3,4.4] | 5.5^***^ [2.7,11.1] | 6.0^***^ [3.1,11.7] |  |
| *CD4 at first visit* |  |  |  |  |
| CD4≤350 | 1 | 1 | 1 |  |
| CD4 between ]350-500] | 12.1^***^ [3.3,45.0] | 0.5 [0.2,1.2] | 0.5 [0.2,1.1] |  |
| CD4>500 | 9.8^***^ [2.7,36.1] | 0.8 [0.4,1.6] | 0.9 [0.5,1.6] |  |
| *On ART at M1* |  |  |  |  |
| No | 1 | 1 | 1 |  |
| Yes | 0.05^***^ [0.0,0.2] | 0.1^***^ [0.1,0.2] | 0.7 [0.4,1.2] |  |

^*^ *p* < 0.05, ^**^ *p* < 0.01, ^***^ *p* < 0.001; aOR: adjusted odds ratio

**Supplemental Table 2: Sensitivity analysis of factors associated with trajectory groups (reference= Group 1: remained in care) when patients who transferred out were considered as retained in care, multivariable analysis (ANRS 12249 TasP trial, n=735)**

|  | **Groups** | | | |
| --- | --- | --- | --- | --- |
| **Covariates– aOR [95% CI]** | **Group 2: exited care then returned** | **Group 3: exited care rapidly** | **Group 4: exited care later** |  |
| *Gender & Social support* |  |  |  |  |
| Female & social support | 1.0 | 1.0 | 1.0 |  |
| Female & no social support | 1.4 [0.5,4.1] | 1.7 [0.8,3.8] | 0.5 [0.2,1.4] |  |
| Male & social support | 3.2^**^ [1.4,7.5] | 1.9 [0.9,3.8] | 1.8 [0.9,3.4] |  |
| Male & no social support | 2.8 [0.8,10.5] | 1.8 [0.7,4.9] | 1.6 [0.6,4.2] |  |
| *Age (years)* |  |  |  |  |
| ≥40 | 1.0 | 1.0 | 1.0 |  |
| 30-39 | 0.9 [0.3,2.4] | 1.0 [0.4,2.3] | 3.2^**^ [1.4,7.2] |  |
| 16-29 | 2.5^*^ [1.1,5.9] | 3.5^***^ [1.8,7.0] | 4.8^***^ [2.2,10.3] |  |
| *Having a regular partner* |  |  |  |  |
| Yes | 1.0 | 1.0 | 1.0 |  |
| No | 3.1^*^ [1.3,7.3] | 1.5 [0.7,3.1] | 1.2 [0.6,2.5] |  |
| *Newly diagnosed at referral* |  |  |  |  |
| No | 1.0 | 1.0 | 1.0 |  |
| Yes | 1.1 [0.3,4.3] | 5.3^***^ [2.6,10.6] | 6.2^***^ [3.2,12.1] |  |
| *CD4 at first visit* |  |  |  |  |
| CD4≤350 | 1.0 | 1.0 | 1.0 |  |
| CD4 between ]350-500] | 11.3^***^ [3.1,41.9] | 0.5 [0.2,1.1] | 0.5 [0.2,1.1] |  |
| CD4>500 | 11.1^***^ [3.1,40.2] | 0.8 [0.4,1.6] | 0.8 [0.4,1.5] |  |
| *On ART at M1* |  |  |  |  |
| No | 1.0 | 1.0 | 1.0 |  |
| Yes | 0.1^***^ [0.0,0.2] | 0.1^***^ [0.1,0.2] | 0.7 [0.4,1.3] |  |

^*^ *p* < 0.05, ^**^ *p* < 0.01, ^***^ *p* < 0.001; aOR: adjusted odds ratio
